# The outer-membrane protein MafA of Neisseria meningitidis constitutes a novel protein secretion pathway specific for the fratricide protein MafB

**DOI:** 10.1080/21505594.2020.1851940

**Published:** 2020-12-14

**Authors:** Jesús Arenas, Laura Catón, Tom van den Hoeven, Vincent de Maat, Juan Cruz Herrero, Jan Tommassen

**Affiliations:** a Section Molecular Microbiology, Department of Biology, Utrecht University, Utrecht, Netherlands; b Unit of Microbiology and Immunology, Faculty of Veterinary, University of Zaragoza, Zaragoza, Spain

**Keywords:** Interbacterial competition, MafA, MafB, *Neisseria*, protein secretion, secretome, toxin

## Abstract

MafB proteins are toxins secreted by *Neisseria* spp. which are involved in interbacterial competition. Their secretion mechanism has so far not been elucidated. Each strain can produce several MafB variants. On the chromosome, the *mafB* genes are localized on genomic islands also containing *mafA* genes. MafA proteins have a role in virulence with reported activities in adhesion and transcytosis of pathogenic *Neisseria, a priori* unrelated to MafB activities. In this study, we investigated the possible involvement of MafA in the transport of MafB across the outer membrane of *Neisseria meningitidis*. In wild-type strains, proteolytic fragments of MafB proteins were detected in the extracellular medium. In the absence of MafA, secretion was abrogated, and, in the case of MafB_I_, full-length and truncated polypeptides were detected inside the cells and inside outer-membrane vesicles. MafB_I_ secretion required its cognate MafA, whereas MafB_III_ could use any MafA. Heterologous expression in *Escherichia coli* showed that MafB_III_ is transported to a cell-surface-exposed, i.e. protease-accessible, location in a MafA-dependent way. MafA itself was found to be localized to the outer membrane, forming large oligomeric complexes. As homologs were found in diverse bacteria, the Maf system represents a new protein secretion system in Gram-negative bacteria.

## Introduction

Proteins produced by Gram-negative bacteria and destined to the extracellular milieu must be translocated across a cell envelope composed of two membranes, the inner membrane (IM) and the outer membrane (OM), which are separated by the periplasm containing a peptidoglycan layer. Various protein secretion systems, dubbed type I to VI, have evolved in these bacteria [1]. Secretion via the type V system occurs in two steps. Proteins first cross the IM using the Sec machinery. Subsequently, they cross the OM via a specific transporter, which can be an independent protein or be part of the secreted protein in the cases of two-partner secretion (TPS) systems [2] and autotransporters [3], respectively. In both cases, the transporter is integrated into the OM by the β-barrel assembly machinery (BAM), a complex of several proteins that orchestrates the folding and insertion of new OM proteins [4]. In the TPS system, the secreted protein and the transporter are generically called TpsA and TpsB, respectively. TpsB is a homolog of BamA, the central component of the BAM. It consists of a 16-stranded OM-embedded β-barrel and two periplasmically exposed polypeptide transport-associated (POTRA) domains [2]. TpsA interacts with TpsB via its N-terminally located TPS domain, which is recognized by the POTRA domains of the TpsB [2]. In several microorganisms, TPS systems are involved in interbacterial competition by inhibiting the growth of related bacteria. In such cases, they are also referred to as CDI (**c**ontact-**d**ependent growth-**i**nhibition) systems [5]. The TpsA (or CdiA) proteins of these systems are very large β-helical protein of >2000 amino-acid residues that display a small toxic domain at the C terminus. In the proposed model [6], surface-exposed TpsA interacts with a receptor on the target cells, e.g. BamA or another OM protein [5], after which the toxic domain of TpsA is cleaved and transported into the cytosol of the target cell, where it exerts one of various toxic activities. A small protein encoded by a gene called *tpsI*, which is located immediately downstream of the *tpsA* gene, protects the producing cells against the toxic C-terminal domain of TpsA; thereby, the C-terminal domain of TpsA and TpsI constitute together a toxin/antitoxin module [5].

*Neisseria meningitidis* is a commensal inhabitant of the upper respiratory tract of humans, but occasionally, it can reach the blood stream and cross the blood-brain barrier to cause sepsis and/or meningitis. The type V secretion system (T5SS) constitutes the main secretion pathway in *N. meningitidis*, where it encompasses up to eight different autotransporters and, dependent on the specific strain, one or several TPS systems [7]. The TPS systems are involved in CDI [8], besides additional functions in biofilm formation and pathogenesis [9]. The genes for the TPS systems are often localized on genomic islands, which usually also contain a repertoire of *tpsC* cassettes. These cassettes putatively encode N-terminally truncated TpsA proteins with sequences for different toxic domains and are associated with genes encoding the corresponding immunity proteins. They can recombine with the *tpsA* locus, thereby replacing the toxic domain of the TpsA protein [8].

**Figure 1.**
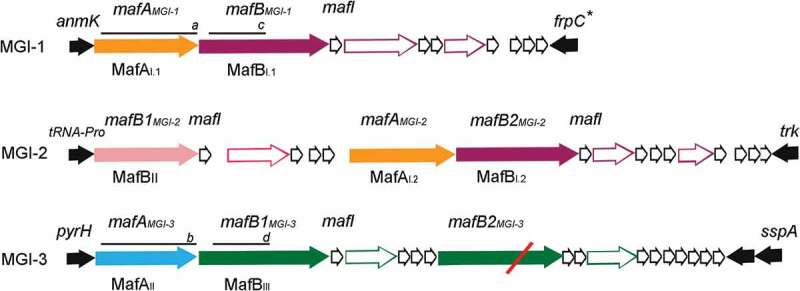
Organization of the MGI of *N. meningitidis* strain B16B6. Strain B16B6 has three MGI [10]. Genes flanking the islands are indicated with filled black arrows. Filled colored arrows indicate *mafA* and *mafB* genes; their proposed systematic names [11] and the phylogenetic classes of the encoded proteins [10] are given above and below the arrows, respectively. Color coding: orange, MafA_I_; blue, MafA_II_; garnet, MafB_I_; pink, MafB_II_; green, MafB_III_. Open colored arrows downstream of the *mafB* genes refer to truncated *mafB* genes that lack regular 5ʹ sequences. Their color refers to high sequence similarity with the upstream intact *mafB*, although their 3ʹ end, encoding the toxic domain, is entirely different. MGI-3 contains an additional complete *mafB* gene, but it is disrupted by a frameshift mutation (indicated with red slash). Open black arrows represent small open reading frames, including *mafI* genes encoding immunity proteins. Where multiple small open reading frames are located downstream of a *mafB* or truncated *mafB*, it is not always clear which one encodes the corresponding immunity protein. Lines numbered *a-d* refer to the polypeptides that were produced to raise antisera

Blast searches using different toxic domains of TpsA proteins as queries identified a novel growth-inhibition system in *Neisseria* spp., the MafB proteins [10,11]. Apart from the presence of the toxic domain, MafB proteins do not show any similarity with TpsA proteins. They consist of an N-terminal signal sequence, a conserved domain of unknown function, i.e. DUF1020, and a C-terminal toxic domain. In some MafB proteins, a Hint domain, which is expected to mediate protein self-splicing [12,13] separates the DUF1020 from the toxic domain. The *mafB* genes are localized on the chromosome in genomic islands designated Maf Genomic Islands (MGI). Up to three MGI can be present in a single meningococcal strain (Figure 1) [11]. Based on phylogenetic analysis of the DUF1020 domain, three classes of MafB can be distinguished, designated MafB_I_-MafB_III_ (Figure 1), where classes I and II MafB proteins are more closely related to each other than to MafB_III_ [10]. Within the MGI, the *mafB* genes are flanked by a *mafI* gene, which encodes an immunity protein that protects the producing cells against the toxic activity of MafB [10,11], and, usually, a *mafA* gene (Figure 1). Phylogenetic analysis distinguished two classes of MafA proteins, MafA_I_ and MafA_II_ [10], where *mafB*_I_ and *mafB*_III_ genes are always associated with *mafA*_I_ and *mafA*_II_ genes, respectively. The *mafB*_II_ genes are not directly adjacent to a *mafA* gene, but they are localized on an MGI also containing a *mafB*_I_ and a *mafA*_I_ (Figure 1). In addition, each MGI contains a variable repertoire of genes putatively encoding N-terminally truncated MafB proteins with variable toxic domains and cognate immunity proteins (Figure 1), an organization resembling the *tpsC* cassettes in the TPS islands.

The secretion mechanism of MafB has not been elucidated so far. MafA would be an obvious candidate for a protein involved in the secretion process, but such a role could not be demonstrated [11]. MafA is produced with a signal sequence typical of lipoproteins (Figure S1A). The protein does not show any similarity with TpsB and, in fact, secondary structure predictions do not suggest a β-barrel structure, which is typical for integral OM proteins, at all (Figure S1A). Previously, MafA proteins have been reported to function as adhesins that bind host glycolipids [14] (hence the notation Maf, which stands for **m**eningococcal **a**dhesin **f**amily). Recently, Káňová et al (2019) showed that meningococcal MafA_I_ protein binds to human brain microvascular endothelial cells (hBMEC) [15], evidencing that the protein participates in the adhesion of the meningococcus to eukaryotic cells. Moreover, transcriptome analysis of hBMEC stimulated with MafA protein revealed that the hBMEC responsed by regulating a large number of genes participating in a variety of biological processes related to bacterial transcytosis [16], including cell surface modifications, endocytosis, extracellular matrix reorganization, and proinflammatory cytokine production, among others. Thus, reported evidences suggest an important role of MafA in the virulence of pathogenic *Neisseria* spp. Nevertheless, we decided to explore further the possibility that MafA is the dedicated transporter of MafB, and the results are presented here.

## Material and methods

### Bioinformatics

Amino-acid sequences encoded by *mafA* and *mafB* genes were predicted using clone Manager Suite 7. The cleavage site of the N-terminal signal sequence of MafB proteins was predicted using (http://www.cbs.dtu.dk/services/SignalP/) and the lipobox of MafA proteins using (http://www.cbs.dtu.dk/services/LipoP/). Protein sequences were aligned in MAFFT version 7 (http://align.bmr.kyushu-u.ac.jp/mafft/online/server/). For secondary structure predictions PsiPred [17] was used. For helical wheel projections HELIQUEST was used (http://heliquest.ipmc.cnrs.fr).

### Bacterial strains and growth conditions

All strains used in this study are listed in Table 1. *N. meningitidis* strain BB-1 is an unencapsulated derivative of B16B6 [18]. Meningococcal strains were grown overnight on GC medium base (Difco) supplemented with IsovitaleX (Becton Dickinson) at 37°C in a candle jar. To grow the bacteria in liquid cultures, bacteria were collected from GC plates and diluted in tryptic soy broth (Beckton Dickinson) to an optical density at 550 nm (OD_550_) of 0.15 and incubated in 25-cm^2^ polystyrene cell culture flasks or 125-ml square media bottles with constant shaking at 110 r.p.m. for 6 h to an OD_550_ of ~2. *Escherichia coli* strains DH5α and BL21(DE3) were used for cloning and protein production, respectively, and they were grown in lysogeny broth (LB) while shaking at 200 r.p.m. at 37°C or on LB plates.

**Table 1.** Bacterial strains and plasmids used in this study

Strains or plasmids	Description^a^	Reference^b^
Strains		
*E. coli*		
DH5α	F^−^, Δ*(lacZYA-argF)U169 thi-1 hsdR17 gyrA96 recA1 endA1 supE44 relA1 phoA Φ80*dlacZΔM15	UU
BL21(DE3) *N. meningitidis*	Contains gene for T7 DNA polymerase	Invitrogen
FAM18	Wild type, C:2a, cc11	
B16B6	Wild type, B:2a, cc11	
BB-1	B16B6 with the capsule locus replaced by *ery*	[18]
BB-1 Δ*mafA*_I.1_	BB-1 with *mafA*_MGI-1_ replaced by *kan*	[10]
BB-1 Δ*mafA*_I.2_	BB-1 with *mafA*_MGI-2_ replaced by *kan*	[10]
BB-1 Δ*mafA*_II_	BB-1 with *mafA*_MGI-3_ replaced by *kan*	[10]
BB-1 Δ*mafB*_I.1_	BB-1 with *mafB* and downstream cassettes of MGI-1 replaced by *kan*	[10]
BB-1 Δ*mafB*_I.2_	BB-1 with *mafB2* and downstream cassettes of MGI-2 replaced by *kan*	[10]
BB-1 Δ*mafB*_I.1_ Δ*mafB*_I.2_	BB-1 with *mafB and mafB2* and downstream cassettes replaced by *kan* and *cat*, respectively	This study
BB-1 Δ*nalP*	BB-1 with *nalP* replaced by kan	[18]
BB-1 Δ*iga*	BB-1 with *iga* replaced *kan*	[18]
BB-1 Δ*app*	BB-1 with *app* replaced by *kan*	[18]
BB-1 Δ*mafA*_I.1_ Δ*mafA_I_*_.2_	BB-1 with *mafA*_MGI-1_ and *mafA*_MGI-2_ replaced by *kan* and *cat*, respectively	This study
	B16B6Δ*maf*A_I.1_ Δ*mafA*_I.2_ Δ*mafA*_II_ B16B6 with *mafA*_MGI-1,_ *mafA*_MGI-2_ and *mafA*_MGI-3_ replaced by *kan, cat* and *gm*, respectively	This study
B16B6 Δ*maf*A_I.1_ Δ*mafA*_I.2_	B16B6 with *mafA*_MGI-1_ and *mafA*_MGI-2_ replaced by *kan* and *cat*, respectively	This study
Plasmids		
pET16b	T7 promoter, 5‘ His-tag sequence in frame, *lacI, amp*	Invitrogen
pET-MafA_I-H_	pET16b plasmid containing partial *mafA*_MGI-1_ gene of BB-1. *amp*	This study
pET-MafA_II-H_	pET16b plasmid containing partial *mafA*_MGI-3_ gene of BB-1. *amp*	This study
pET-MafB_I-H_	pET16b plasmid containing partial *mafB*_MGI-1_ gene of BB-1. *amp*	This study
pET-MafB_III-H_	pET16b plasmid containing partial *mafB*1_MGI-3_ gene of BB-1. *amp*	This study
pIN_L_	phrtA-gm-gfp containing a constitutive *opaBP*_L_ promoter. *gm, kan, amp*	[19]
pIN-A_II_-B_III_-I	Derivative of pIN_L_ containing *mafA*_II,_ *mafB*_III_ and *mafI* of BB-1. *amp, kan, gm*	This study
pIN-B_III_-I	Derivative of pIN_L_ containing *mafB*_III_ and *mafI* of BB-1. *amp, kan, gm*	This study
pFPIORF_1_	pEN plasmid containing IORF_1_ gene of BB-1. *ery, cat*	[8]
pEN-*mafB*_I-H_	pEN plasmid containing partial *mafB*_I.1_ gene of FAM18 with His tag sequence. *ery, cat*	This study
pEN-*mafB*_I-S_	pEN plasmid containing partial *mafB*_I.1_ gene of FAM18 with Strep tag sequence. *ery, ca*	This study
pEN-*mafB*_III-S_	pEN plasmid containing partial *mafB*_III_ gene of BB-1 with strep tag sequence. *ery, cat*	This study
pEN-*mafA*_I-H_	pEN plasmid containing *mafA*_I.1_ gene of BB-1 with His tag sequence. *ery, cat*	This study
pEN-*mafA*_II-H_	pEN plasmid containing *mafA*_II_ gene of BB-1 with His tag sequence. *ery, cat*	This study
pKO*mafA*_MGI-2_::*kan*	*mafA*_I.2_ knockout construct. *amp, kan*	[10]
pKO*mafA*_MGI-3_::*kan*	*mafA*_II_ knockout construct. *amp, kan*	[10]
pKO*mafA*_MGI-2_::*cat*	pKO*mafA*_MGI-2_::*kan* derivative with *kan* replaced by *cat. amp, cat*	This study
pKO*mafA*_MGI-3_::*gm*	pKO*mafA_MGI-3_::kan* derivative with *kan* replaced by *gm. amp, gm*	This study
pKO*nhbA*-cat	*nhbA* knockout construct. *amp, cat*	[18]

^a^*kan*, kanamycin-resistance cassette; *amp*, ampicillin-resistance cassette; *gm*, gentamicin-resistance cassette; *cat*, chloramphenicol-resistance cassette; *ery*, erythromycin-resistance cassette; cc, clonal complex. ^b^UU, Utrecht University laboratory collection.

For plasmid maintenance, the culture medium was supplemented where appropriate with antibiotics in the following concentrations: 7 µg ml^−1^ chloramphenicol (Sigma-Aldrich), 7 µg ml^−1^ erythromycin (Fluka Analytical), 100 µg ml^−1^ kanamycin (Sigma-Aldrich), or 60 µg ml^−1^ gentamicin (Sigma-Aldrich) for *N. meningitidis*, and 25 µg ml^−1^ chloramphenicol, 150 µg ml^−1^ erythromycin, 100 µg ml^−1^ kanamycin, or 20 µg ml^−1^ gentamicin for *E. coli*. To induce gene expression, 0.1 mM isopropyl-β-D-1-thiogalactopyranoside (IPTG) (Thermo Scientific) was added at the start of the liquid cultures.

### DNA constructs

DNA fragments were amplified by PCR from chromosomal DNA of *N. meningitidis* strains BB-1 and FAM18 with primers listed in Supplementary Table S1 using High Fidelity Polymerase (Roche Diagnostics GmbH, Germany) or DreamTaq-DNA Polymerase (Fermentas, UK). PCR products were visualized in 1% agarose gels with ethidium bromide. Plasmids used in this study are listed in Table 1. For cloning, PCR products and vectors were purified using the Clean-Up System and Plasmid Extraction kit, respectively (both from Promega). Purified vectors and PCR products were digested with the restriction enzymes (Fermentas) for which sites were included in the primers (Table S1) and subsequently ligated together. *E. coli* DH5α was transformed with ligation products, and transformants were selected on LB- agar plates supplemented with appropriate antibiotics. Correct clones were elected by PCR, and plasmids were purified and sequenced at the Macrogen sequencing service (Amsterdam). Then, plasmids were transferred by transformation to *E. coli* BL21(DE3) or to *N. meningitidis* strains. For *E. coli*, 5 µl of plasmid was added to competent cells suspended in LB medium supplemented with 85 mM of CaCl_2_ and 15% of glycerol (v/v), incubated 15 min on ice, and the bacterial suspension was placed during 30 seconds at 42ºC, and immediate after incubated on ice during 5 min. Then, bacterial cells were resuspended in LB medium and incubated at 37ºC at 200 r.p.m during 1 h. Finally, cells were harvested by centrifugation and spread onto agar plates supplemented with appropriated antibiotics. For *N. meningitidis*, an overnight colony was picked up and streak onto a small area (~2 cm^2^) of a GC-agar plate, and 15 µl of purified plasmid in 10 mM of MgCl_2_ was dropped on top of the culture and allowed to air-dry at room temperature. After 8 h of incubation at 37ºC, the colony was spread onto an GC-agar plate supplemented with appropriated antibiotics.

To generate constructs for antiserum production, DNA fragments corresponding to amino-acid residues 16–313 of MafA_I_, 21–320 of MafA_II_, 28–239 of MafB_I.1_, and 26–239 of MafB_III_ (fragments *a-d* in Figure 1) were amplified from BB-1. These fragments are missing the sequences encoding the signal sequences and, for the MafB proteins, the C-terminal toxic domains. They were cloned into pET16b, thereby generating an N-terminal His-tag in the produced proteins. The resulting plasmids were called pET-MafA_I-H_, pET-MafA_II-H_, pET-MafB_I-H_ and pET-MafB_III-H_, respectively. To generate plasmids for the synthesis of MafB_III_ and MafI from MGI-3 either with or without the cognate MafA_II_ in *E. coli*, PCR fragments were cloned into pIN_L_, a derivative of pCRII-TOPO carrying a modified *opaB* promoter [19]. The resulting plasmids are called pIN-A_II_-B_III_-I and pIN-B_III_-I, respectively. To produce recombinant MafB_I_ (NMC1790) of FAM18 and MafB_III_ of BB-1 in *N. meningitidis*, PCR products containing the *mafB* genes without the sequences corresponding to the toxic domain were cloned into plasmid pFPIORF_1_ [8]. Sequences for a Strep- or a 10xHis-tag were incorporated in the reverse primers (Table S1). The corresponding plasmids were called pEN-*mafB*_I-S,_ pEN-*mafB*_I-H_ and pEN-*mafB*_III-S_, where the affixes S and H refer to the presence of a Strep- or a His-tag, respectively. The *mafA* genes from MGI-1 and MGI-3 were amplified from BB-1 with sequences for a 6xHis-tag sequence included in the reverse primer and cloned into pFPIORF_1_. The resulting constructs were called pEN-*mafA*_I-H_ and pEN-*mafA*_II-H_, respectively. The synthesis of all recombinant proteins was detected by Western blotting.

For the construction of double and triple *mafA* mutants, the kanamycin-resistance cassettes of the *mafA*_MGI-2_ and *mafA*_MGI-3_ knockout constructs [10] were replaced by a chloramphenicol-resistance cassette from pKO*nhbA*-cat [8] and a gentamicin-resistance cassette amplified by PCR from pIN_L_ [19], respectively, via SalI digestion.

### Cell fractionation, SDS-PAGE and Western blotting

*N. meningitidis* and *E. coli* strains were grown to an OD_550_ of 2 and an OD_600_ of 1, respectively. The cultures were centrifuged (4,500 *g* for 5 min), and the resulting cell pellet was resuspended in H_2_O to a final OD_550_ of 10. The spent media were centrifuged at 16,000 *g* for 15 min to remove residual cells, and proteins were precipitated from the supernatants with 10% (w/v) ice-cold trichloroacetic acid (TCA) in H_2_O. For SDS-PAGE and Western-blot analysis, supernatant samples were 50-fold concentrated relative to the cell samples. OM vesicles (OMVs) were removed from spent medium fractions by ultracentrifugation at 100,000 *g* for 2 h, and proteins were precipitated from the resulting supernatant with TCA as above. The OMVs were resuspended in H_2_O in the same volume as that of the supernatant fraction. For cell envelope isolation, bacteria cells from 6-h old growth cultures, were harvested by centrifugation and resuspended in 50 mM Tris-HCL with 5 mM EDTA (pH 8.0) and stored overnight at −20ºC. Cells were disrupted by sonication with a Branson sonifier 450 (Brason Ultrasonics Corporation) three times during 45 s (level 8, output 40%). Unbroken cells were first removed by centrifugation (12,000 *g* for 30 min at 4ºC). Cell envelopes, present in the supernatant, were collected by ultracentrifugation (170,000 × *g* for 30 min at 4°C), resuspended in 2 mM Tris-HCl (pH 7.6), and stored at −20°C. Protein concentrations in cell envelope preparations were determined with the BCA assay kit (Thermo Fisher Scientific). SDS-PAGE was performed on 8, 12 or 14% polyacrylamide gels in a discontinuous buffer system. Before loading, samples were mixed 1:1 with double-strength sample buffer [0.125 M Tris-HCl, pH 6.8, 20% (v/v) glycerol, 4% (w/v) SDS, 0.02% bromophenol blue, 5% (v/v) β-mercaptoethanol] and heated for 10 min at 100°C. For semi-native SDS-PAGE, the sample buffer contained only 0.2% SDS (final concentration) and no β-mercaptoethanol, and the samples were kept at room temperature before electrophoresis [20]. Electrophoresis was carried out at 200 V for ~45 min at room temperature or at 12 mA for ~180 min on ice for semi-native SDS-PAGE. Proteins separated on gels were stained with Coomassie brilliant blue G250 or transferred to nitrocellulose membranes, and the Western blots were developed as previously described [18]. The monoclonal antibody MN2D6D directed against RmpM was generously provided by the Netherlands Vaccine Institute (Bilthoven, The Netherlands), and the DsbA1 antiserum was previously described [21]. Monoclonal anti-His (Sigma Aldrich) and anti-Strep (Novagen) antibodies were also used. The antisera directed against the α-peptide and the translocator domain of IgA protease [22] and monoclonal antibody 2CE directed against OmpA were from our laboratory collection.

### Purification of recombinant Maf proteins and antiserum production

MafA and fragments of MafB proteins of strain BB-1 were produced and purified as described [18]. Briefly, the recombinant polypeptides with an N-terminal His-tag were produced from plasmids pET-MafA_I-H_, pET-MafA_II-H_, pET-MafB_I-H_, and pET-MafB_III-H_ in *E. coli* BL21(DE3) after induction with IPTG and purified as inclusion bodies. The protein bands corresponding to the recombinant proteins were excised from SDS-PAGE gels and eluted, and the protein concentration was determined with the BCA assay kit. Purified proteins were used to raise antisera in mice at Eurogentec (Liège, Belgium).

### Protein identification

For MALDI-TOF peptide map fingerprinting analysis, cell envelopes of BB-1 overproducing MafA_II_ from plasmid pEN-*mafA*_II-H_ and, as a control, of its derivative B16B6 Δ*maf*A_I.1_ Δ*mafA*_I.2_ Δ*mafA*_II_ lacking all *mafA* genes were used. Proteins were separated by semi-native SDS-PAGE and individual bands, from the position corresponding to that of MafA_II_ in the overproducing strain as detected on Western blot, were cut from Coomassie brilliant blue G250-stained gels. The excised bands from both samples were sent to the Section of Proteomics (SCSIE) of the Universidad de Valencia (Spain) for protein identification. Samples were digested with sequencing grade trypsin (Promega) as described [23]. The digestion mixtures were dried, resuspended in 7 ml of 0.1% trifluoroacetic acid (TFA), and 1 ml was spotted on a MALDI plate. After the droplets were air-dried at room temperature, 1 μl of matrix [5 mg/ml CHCA (Sigma) in 0.1% TFA-acetonitrile/H_2_O (1:1, v/v)] was added and allowed to air-dry at room temperature. The resulting mixtures were analyzed in a 5800 MALDI TOF/TOF (ABSciex) in positive reflectron mode (3000 shots every position). Five of the most intense precursors (according to the threshold criteria: minimum signal-to-noise: 10; minimum cluster area: 500; maximum precursor gap: 200 ppm; maximum fraction gap: 4) were selected for every position for the MS/MS analysis. MS/MS data was acquired using the default 1kV MS/MS method. The MS and MS/MS information was sent to MASCOT version 2.3.02 via the Protein Pilot version 4.5 (ABSciex). Significant protein scores greater than 83 (P < 0.05) were considered.

### Proteinase K accessibility assays

Proteinase K accessibility of proteins in intact cells was assessed as described [20] with some modifications. Briefly, *N. meningitidis* and *E. coli* from cultures grown to an OD_550_ of 2.0 or an OD_600_ of 0.5, respectively, were adjusted to an OD_550_ of 1 or an OD_600_ of 5, respectively, and incubated with proteinase K (Fermentas) for 1 h at 37°C. The protease was inactivated with 2 mM phenylmethylsulfonyl fluoride (Sigma-Aldrich) for 20 min at room temperature, and cells were harvested by centrifugation. Protease accessibility of proteins in OMV-containing supernatant fractions was assessed by incubating these fractions for 1 h with proteinase K, and protease-resistant proteins were subsequently precipitated with TCA as described above. Protein degradation was examined by SDS-PAGE and Western blotting.

### Separation of OM and IM

OM and IM of *N. meningitidis* were separated by sucrose density gradient centrifugation as described [21] with modifications. Bacteria from liquid cultures grown to an OD_550_ of 1 were inactivated with 1 mg ml^−1^ of streptomycin for 1 h, harvested by centrifugation (4,500 g for 20 min at 4°C), washed in 50 mM Tris-HCl, 5 mM EDTA pH 7.4 (TE), and resuspended in TE buffer with 15% (w/v) sucrose supplemented with 50 µg ml^−1^ of DNase I and RNase A, 0.5 mg ml^−1^ of lysozyme, and 150 mM DL-dithiothreitol (DTT) (all from Sigma-Aldrich). Cells were disrupted in a French pressure cell at 1.5 × 10^5^ kPa. Residual cells were removed by centrifugation (4,000 *g* for 30 min at 4°C), and supernatant was ultracentrifuged (100,000 *g* for 90 min at 4°C) to pellet the cell envelopes, which were resuspended in TE buffer with 15% (w/v) sucrose and 2 mM DTT. This suspension was loaded on top of a discontinuous sucrose gradient consisting of 2.5-ml layers of 55, 50, 45, 40 and 35% (w/w) sucrose, and 1.8 ml of 30% (w/w) sucrose in TE buffer with 2 mM DTT and centrifuged in a Optima LE-80 K ultracentrifuge (25,000 r.p.m. for 16 h at 4°C, Beckman SW28.1 rotor). Fractions of 1 ml were collected from the bottom to the top using a peristaltic pump, and samples were prepared for SDS-PAGE analysis.

## Results

### Secretion and processing of MafB_I_ proteins

**Figure 2.**
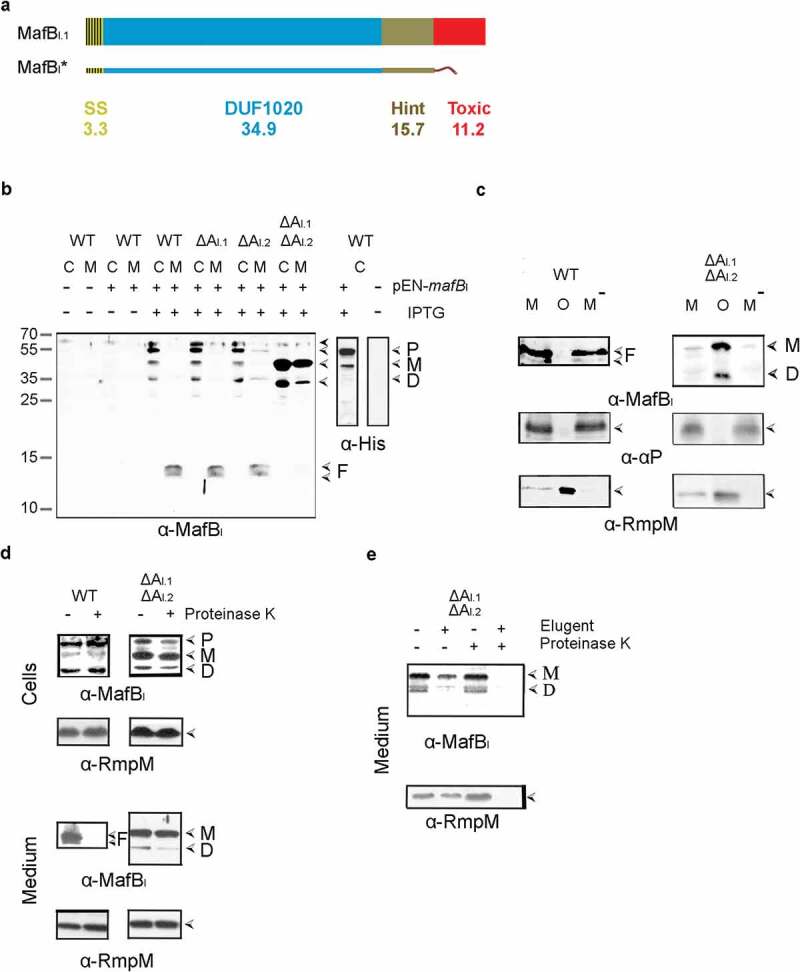
Expression, secretion, and proteolysis of MafB_I_*. (a) Structure of MafB_I.1_ encoded by locus NMC1790 of strain FAM18. The protein contains a signal sequence (SS), a DUF1020 domain, a Hint domain, and a C-terminal toxic domain. The mass of each domain is given (in kDa). The recombinant MafB_I_* proteins lack the toxic domain and instead contain either a Strep-tag or, where explicitly indicated, a His-tag. (b) Secretion and processing of MafB_I_*. Proteins in cell lysates (c) and spent medium (m) of BB-1 (WT) and its single and double *mafA*_I_ mutant derivatives (ΔA_I.1_ and ΔA_I.2_) were separated by SDS-PAGE and probed on Western blots with antisera directed against MafB (α-MafB_I_) or against the His-tag (α-His). The presence or absence in the strains of pEN-*mafB*_I-S_ encoding MafB_I_* with a Strep-tag in the left panel or of pEN-*mafB*_I-H_, which encodes a His-tagged variant of MafB_I_*, in the right panel and of IPTG to induce MafB_I_* production, is indicated. Open arrow-heads indicate MafB forms suggested to be the precursor (p), the mature form after removal of the signal sequence (M), the DUF1020 domain (d), and proteolytic DUF1020 fragments (f). The filled arrow-head indicates a cross-reacting band. (c) The medium fractions (M) of the wild-type and the *mafA*_I_ double mutant, both expressing MafB_I_*, were subjected to ultracentrifugation, and the pelleted OMVs (o) and the OMV-depleted supernatant (M^−^) were analyzed by SDS-PAGE and Western blotting. As controls, the presence in the fractions of the OM-associated periplasmic protein RmpM and the secreted α-peptide (αP) of IgA protease were analyzed. (d) Protease accessibility of MafB_I_* forms. Upper panels: Intact cells of the parent strain (WT) and the Δ*mafA*_I_ double mutant, both synthesizing MafB_I_* from pEN-*mafB*_I-S_, were incubated with 2 µg ml^−1^ of proteinase K. Lower panels: spent-medium fractions of the same cultures were treated with proteinase K. Degradation of various proteins was assessed by Western blotting. Different MafB_I_* forms are indicated at the right and labeled as in panel B. (e) The medium fraction of the Δ*mafA*_I_ double mutant producing MafB_I_* was either treated or not with 1% elugent before proteinase K digestion

To investigate the possible role of MafA proteins in the secretion of MafB proteins, we used *N. meningitidis* strain BB-1, which can potentially synthesize four MafB and three MafA proteins (Figure 1). First, the expression and localization of MafB_I_ proteins was studied. Unfortunately, an antiserum raised against an N-terminal fragment of the MafB_I.1_ protein failed to detect any protein bands on Western blots that could correspond to either one of the two MafB_I_ proteins of BB-1, possibly because the production levels in the growth medium used were too low. Thus, we decided to overproduce a recombinant MafB_I_ from a plasmid, for which we chose MafB_I.1_ encoded on MGI-1 of strain FAM18 (locus tag NMC1790). In contrast to the MafB_I_ proteins of BB-1, this MafB_I_ protein contains a Hint domain in between the DUF1020 and the C-terminal toxic domain (Figure 2(a) and S2). To avoid toxicity problems, the toxic domain of the protein was replaced by a Strep-tag, and the recombinant protein was designated MafB_I_* (Figure 2(a)). IPTG-induced production of MafB_I_* in strain BB-1 resulted in the detection of bands of ~54, 51 and 35 kDa with the anti-MafB_I_ serum in cell lysates, presumably corresponding with the unprocessed precursor of the recombinant protein, the processed full-length protein lacking the signal sequence, and the DUF1020 domain alone, respectively (Figure 2(b), left panel). However, our Strep-tag antibodies did not react with any of these bands. Therefore, to confirm our interpretation of the bands, the Strep-tag was replaced by a His_10_-tag. In this case, we detected the 54- and 51-kDa bands but not the 35-kDa band in cell lysates with anti-His-tag antibodies (Figure 2(b), right panel), confirming that the two larger bands contain an intact C terminus. Remarkably, two prominent bands of 12 and 13 kDa were detected in the culture medium with the anti-MafB_I_ antiserum (Figure 2(b), left panel), indicating that MafB_I_ is secreted and further proteolyzed into fragments. No bands were detected in the culture medium with the anti-His-tag antibodies.

To investigate the role of MafA in the secretion process, the MafB_I_* protein was produced in various *mafA* mutants. Deletion of both *mafA*_I_ genes (orange in Figure 1) individually did not affect the band pattern in cell lysates or spent media as compared with the wild type. However, deletion of both *mafA*_I_ genes together resulted in a strong accumulation of the 51- and 35-kDa forms of MafB_I_* in the cells and a drastic reduction of the smaller fragments in the culture medium (Figure 2(b), left panel). The culture medium, however, also contained considerable amounts of the 51- and 35-kDa forms of MafB_I_*. We considered the possibility that these proteins were associated with spontaneously released OMVs. Indeed, like the OM-associated protein RmpM, these proteins were pelleted from the culture media by ultracentrifugation (Figure 2(c), right panel). In contrast, the 12- and 13-kDa proteolytic fragments detected in the culture medium of the wild-type strain were not removed by ultracentrifugation (Figure 2(c), left panel). Similarly, the α-peptide of the autotransporter IgA protease, which is released from the bacterial cell surface into the culture medium by the proteolytic activity of another autotransporter, i.e. NalP [22], was found in the OMV-depleted culture supernatant (Figure 2(c)). Thus, the 12- and 13-kDa proteolytic fragments of MafB present in the culture supernatant of the wild-type strain are not associated with OMVs.

To further localize the MafB_I_* forms, we assessed their proteinase K accessibility in intact cells and in the OMVs present in the culture media. Treatment of intact cells with proteinase K revealed that the various MafB_I_* forms detected in cell lysates of the wild type and the Δ*mafA*_I_ double mutant were not accessible to the protease (Figure 2(d), top panels), indicating that they are not exposed at the surface of the cells. In contrast, the 12- and 13-kDa MafB_I_* fragments in the culture medium of the wild type were readily degraded (Figure 2(d), bottom left panel) indicating that they were released into the culture medium, while the larger polypeptides associated with the OMVs of the Δ*mafA*_I_ double mutant were inaccessible (Figure 2(d), bottom right panel) and only degraded after solubilization of the OMVs with detergent (Figure 2(e)). Therefore, these polypeptides are encapsulated within the OMVs. As a control, also the OM-associated protein RmpM, which has a large, periplasmically exposed peptidoglycan-binding domain [24] was analyzed. As expected, this protein was only degraded after treatment of the medium fraction with detergent (Figure 2(e)). In conclusion, MafB_I_* is transported across the OM in wild-type cells, and MafA_I_ is involved in this process. At the cell surface, MafB_I_* is proteolyzed generating N-terminal fragments of 12 and 13 kDa, which are released into the milieu. The accumulation of larger forms of MafB_I_* in wild-type cells is probably due to the overproduction of the protein, which cannot be sufficiently dealt with by the amounts of MafA_I_ produced at wild-type level from the chromosome. It is noteworthy that the Δ*mafA*_I_ double mutant used in the experiments described above still contains an intact *mafA*_II_ gene located on MGI-3 (blue in Figure 1), which is apparently not used for the secretion of the recombinant MafB_I_*, thus showing specificity of the transporter.

To further investigate the proteolysis of MafB after secretion, we introduced the plasmid encoding MafB_I_* in different mutants that lack the secreted proteases NalP, IgA protease or App [22,25]. However, all these mutants showed a similar degradation pattern of MafB (Figure S3).

### Secretion and processing of MafB_III_ protein

**Figure 3.**
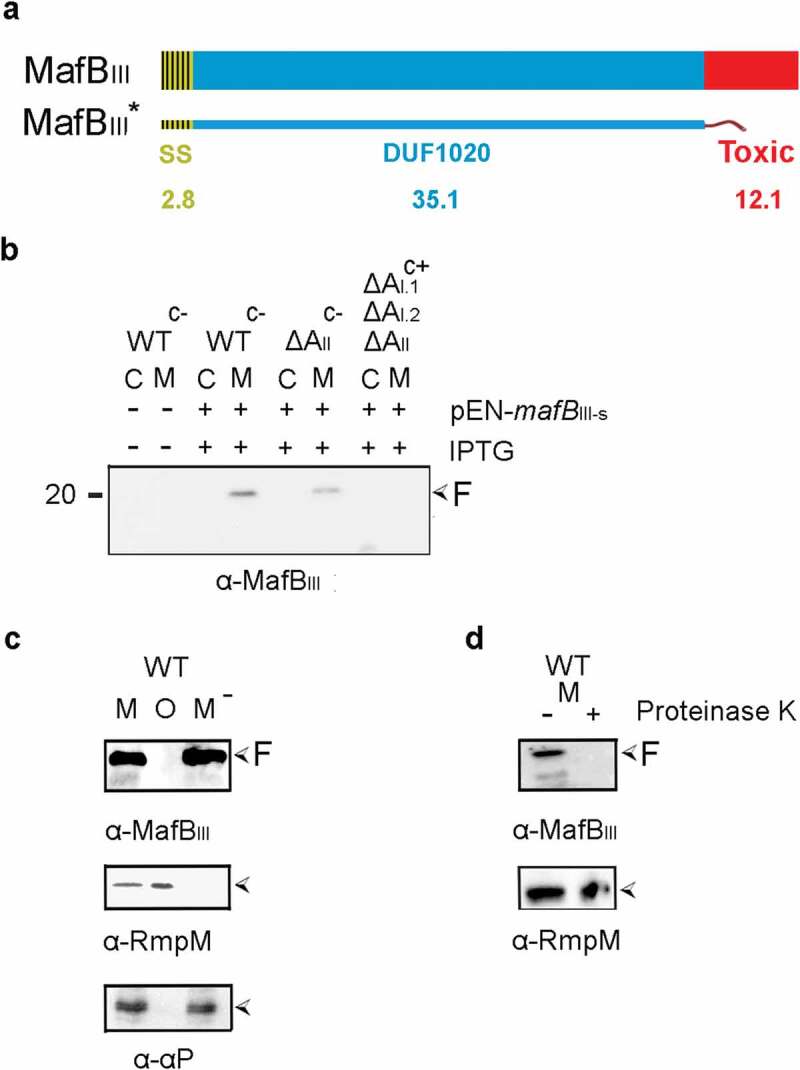
Expression, secretion, and proteolysis of MafB_III_. (a) Structure of MafB_III_ encoded on MGI-3 of strain B16B6. The protein contains a signal sequence (SS), a DUF1020 domain, and a C-terminal toxic domain. The mass of each domain is given (in kDa). The truncated MafB_III_* protein lacks the toxic domain and instead contains a Strep-tag. (b) Expression and localization of MafB_III_* in *N. meningitidis*. Proteins in cell lysates (c) and spent medium (m) of the unencapsulated strain BB-1 (WT^c-^), its derivative lacking *mafA*_II_ (ΔA_II_), and a derivative of the encapsulated parental strain B16B6 (^c+^) lacking all *mafA* genes (ΔA_I.1_ΔA_I.2_ΔA_II_) were separated by SDS-PAGE and probed on Western blots with antiserum directed against MafB_III_. The presence of plasmid pEN-*mafB*_III-S_ in the strains and of IPTG during growth is indicated. (c) The medium fraction (M) of strain BB-1 expressing MafB_III_* was subjected to ultracentrifugation, and the pellet containing OMVs (o) and the OMV-depleted supernatant (M^−^) were analyzed by SDS-PAGE and Western blotting. As controls, the presence in the fractions of the OM-associated protein RmpM and the secreted α-peptide (αP) of IgA protease was analyzed. (d) Culture medium from strain BB-1 expressing MafB_III_* was treated with 2 µg ml^−1^ proteinase K, and degradation of MafB_III_* and, as a control, of the OM-associated periplasmic protein RmpM, was assessed by Western blotting. In panels b-d, F indicates proteolytic fragments of MafB_III._

We next analyzed the synthesis and localization of MafB_III_ protein (green in Figure 1). MafB_III_ protein consists of a DUF1020 domain and a C-terminal toxic domain without a Hint domain in between (Figure 3(a) and Figure S2). MafB_I_ of FAM18 and MafB_III_ of B16B6 or FMA18 are very different; they only share 60% identity in a 70 amino-acid long region within the DUF1020 domain (Figure S2). An antiserum raised against an N-terminal fragment of the protein failed to detect a band that could correspond with MafB_III_ in cells or spent culture medium of BB-1. Thus, a plasmid was constructed to produce a recombinant MafB_III_ protein, designated MafB_III_*, which lacks the C-terminal toxic domain (Figure 3(a)), and the plasmid was introduced into strain BB-1. Upon induction with IPTG, a prominent band of 20 kDa was detected in the spent medium with the anti-MafB_III_ serum on Western blots (Figure 3(b)). Like the secreted α-peptide of IgA protease and unlike the OM-associated protein RmpM, this band was not pelleted with the OMVs (Figure 3(c)), and it was sensitive to proteinase K (Figure 3(d)), demonstrating that it was released into the external medium. Unexpectedly, deletion of the *mafA*_II_ gene located on MGI-3 (blue in Figure 1) did not affect the presence of the band in the medium fraction (Figure 3(b)), suggesting that the secretion of MafB_III_ might also be mediated by MafA_I_ proteins (orange in Figure 1). To test this possibility, a triple mutant was constructed that lacks all *mafA* genes. This was done in the encapsulated parental strain of BB-1, i.e. strain B16B6, to have the availability of enough antibiotic-resistance markers for the gene disruptions. The abundance of the 20- kDa MafB_III_-specific band in the medium fraction was similar in the wild-type B16B6 strain as in the unencapsulated BB-1 strain (data not shown). Indeed, deletion of all *mafA* genes resulted in a drastic reduction in the amounts of the 20-kDa MafB_III_-specific band in the medium (Figure 3(b)). However, we still detected the presence of the MafB_III_ fragment in the triple mutant when our blot was overexposed (Figure S4). Even in this overexposure, no MafB_III_-specific bands were detected in the cell lysates (Figure S4), indicating that MafB_III_ is intracellularly degraded when it is not secreted. Thus, MafB_III_ secretion can apparently be mediated by different MafAs. Together, these results indicate that MafB_III_ can be secreted not only by the MafA_II_ encoded on the same MGI but also via the MafA_I_ proteins encoded on the other MGIs. The minor amounts of MafB_III_ fragment still detectable in the supernatant of the triple *mafA* mutant may result from secretion via a bypass pathway or from cell lysis. Its presence indicates that the proteolysis generating this fragment is not dependent of MafA expression.

### Reconstitution of MafB_III_ secretion in E. coli

**Figure 4.**
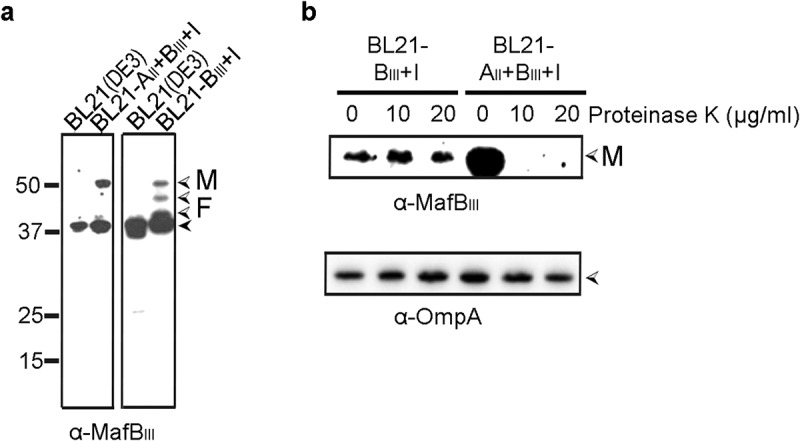
Expression and localization of MafB_III_ in *E. coli*. (a) MafB_III_ and the corresponding MafI were produced in BL21(DE3) either with (BL21-A_II_+B_III_+I) or without (BL21-B_III_+I) MafA_II_, and cell lysates were analyzed by Western blotting with antiserum against MafB_III_. The blot at the right was exposed longer than the one at the left to obtain visible MafB-specific signals. (b) Intact cells of both BL21(DE3) derivatives were incubated with the indicated concentrations of proteinase K and degradation of MafB_III_, and, as a control, of OmpA, a β-barrel OM protein with a large periplasmic domain, was assessed by Western blotting. In both panels, M indicates the full-length mature form of MafB_III_, and F in panel A indicates proteolytic fragments of the protein. The filled arrow-head in panel A indicates a background band

To confirm the MafA-dependency of MafB_III_ secretion and to investigate whether MafA is sufficient for this process, we attempted to reconstitute MafB_III_ secretion in *E. coli*. Two DNA fragments, one containing the entire *mafA, mafB* and *mafI* genes and the other containing only *mafB* and *mafI* of island MGI-3, were cloned into a plasmid, and MafB production and secretion were assessed in *E. coli* strain BL21(DE3). In both cases, intact MafB_III_ was detected in whole cell lysates (Figure 4(a)) and not in the culture supernatants. When MafB_III_ was expressed without MafA_II_, the band corresponding to the full-length MafB_III_ was of lower intensity, and bands of lower apparent molecular weight, probably corresponding to degradation products, were detected. To determine the surface exposure of the proteins, their proteinase K accessibility in intact cells was assessed. MafB_III_ was degraded by proteinase K but only when it was co-expressed with MafA_II_ (Figure 4(b)). The OM protein OmpA, which has a large C-terminal periplasmic domain, was not degraded by proteinase K showing the integrity of the OM. Thus, these data confirm that MafA mediates the transport of MafB_III_ to the bacterial cell surface and that no other specific protein from *N. meningitidis* is required for this secretion process.

### Expression and localization of MafA proteins

**Figure 5.**
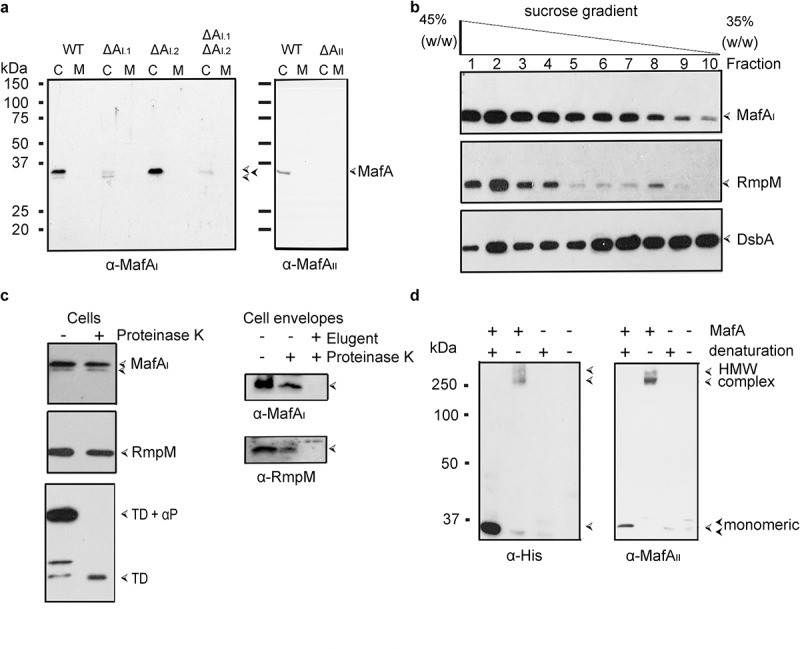
Synthesis and subcellular localization of MafA. (a) The synthesis and possible secretion of MafA proteins were examined by analyzing cell lysates (c) and spent media (m) of BB-1 (WT) and various Δ*mafA* mutant derivatives on Western blots with anti-MafA antisera. The positions of MafA proteins are indicated with open arrowheads. A faint cross-reactive band with a similar electrophoretic mobility is visible in the *mafA*_I.1_ mutant and the double mutant and is indicated with a black arrowhead. (b) Membranes of BB-1 synthesizing a His-tagged recombinant MafA_I_ protein from plasmid pEN-*mafA*_I-H_ were separated by sucrose density gradient centrifugation, and the fractions obtained were analyzed by Western blotting with antibodies directed against the His-tag, against RmpM as an OM marker, and against DsbA as IM markers. (c) Intact cells or cell envelopes of BB-1Δ*nalP* expressing His-tagged MafA_I_ were treated with 2 µg ml^−1^ proteinase K as indicated. Cell envelopes were treated or not with 1% elugent before proteinase K digestion. After incubation, the degradation of MafA_I_, RmpM and the α-peptide (αP) of IgA protease were assessed by Western blotting using specific antibodies. The αP is exposed at the cell surface covalently connected to the translocator domain (TD) of IgA protease [22]. For this blot, an antiserum directed against the TD was used. (d) Western blot analysis of cell envelopes from strain BB-1 synthesizing or not His-tagged MafA_I_ from plasmid pEN-*mafA*_I-H_ and MafA_II_ from the chromosome. The blots were probed with anti-His-tag antibodies and anti-MafA_II_ antiserum, respectively. Before electrophoresis, proteins were either denatured or not by heating the samples at 95°C. HMW complex indicates the position of the high-molecular-weight complexes of the MafA proteins detected in the unheated samples. Filled arrowheads mark weak cross-reactive bands. Numbers at the left indicate the positions of molecular mass markers when required. In panels b and c, only relevant parts of the blots are shown

To mediate the secretion of MafB, MafA should be localized in the OM. To confirm its subcellular localization and study its membrane topology, antisera were raised against MafA_I.1_ and MafA_II_ proteins of BB-1. Both mature MafA_I_ proteins (orange in Figure 1) differ in only two amino-acid residues (Figure S1b) and, therefore, cross-reactivity is to be expected. They share 61% of sequence identity with MafA_II_ (blue in Figure 1; Figure S1b). Two bands with apparent molecular weights of ~34 kDa, corresponding with the expected mass of MafA proteins, were detected on Western blots with the anti-MafA_I_ serum in whole cell lysates of the parental strain (Figure 5(a), left panel). The upper and most prominent band was not present in the lysates of the Δ*mafA*_I.1_ mutant and thus corresponds with MafA_I.1_, whilst the lower and fainter band was not detected in the Δ*mafA*_I.2_ mutant (Figure 5(a), left panel). The anti-MafA_II_ antiserum detected only one band of approximately 34 kDa in whole cell lysates of BB-1, and this band was absent in the Δ*mafA*_II_ mutant (Figure 5(a), right panel). No MafA-related bands were detected in the spent culture media (Figure 5(a)).

Upon cell fractionation, the MafA proteins were detected in the cell envelope fraction (shown for MafA_I.1_ in Figure S5). To localize them more precisely, the OM and IM were separated by sucrose density gradient centrifugation. Since the antibodies hardly detected the proteins in the separate fractions, recombinant His_6_-tagged MafA_I.1_ and MafA_II_ were used in these experiments. MafA_I.1_ co-fractionated with the OM-associated marker protein RmpM and clearly different from the DsbA lipoproteins, which are two IM-associated lipoproteins in *N. meningitidis* [21] (Figure 5(b)). To test whether the protein is surface exposed, its proteinase K accessibility was assessed. For these experiments, we used a *nalP* mutant of strain BB-1. In this strain, the α-peptide of IgA protease remains present at the cell surface associated with the translocator domain of the autotransporter [22], allowing its use as a protease-accessible positive control. In contrast to the α-peptide, MafA_I.1_, MafA_I.2_ and MafA_II_ were not affected by treatment of intact bacteria with proteinase K (shown for the MafA_I_ proteins in Figure 5(c), left panel). However, similar as the periplasmically exposed OM-associated protein RmpM, MafA was partially degraded by treatment of cell envelopes with proteinase K and completely when the cell envelopes were first solubilized in detergent before protease treatment (Figure 5(c), right panels). Together, these results demonstrate that MafA is located in the OM and accessible for proteases from the periplasmic side, but it does not expose large protease-accessible loops at the cell surface.

MafA is not predicted to form a β-barrel structure as typical OM proteins do (Figure S1a). Thus, we asked how MafA could be integrated in the OM to be able to function as a transporter. Some transporters for macromolecules in the OM, such as the capsular polysaccharide transporter Wza [26] or the VirB10 component of the type IV secretion system [27], form multimers with each monomer inserted into the OM only with a small segment of the polypeptide, and together, these small segments are forming a central channel. To investigate the possibility that also MafA forms multimers, cell envelope preparations were analyzed by SDS-PAGE either with or without heat denaturation of the samples before electrophoresis. The anti-MafA_I_ serum did neither detect monomeric nor multimeric forms of MafA_I_ in non-heated samples, suggesting that the epitopes are masked when the protein is in its native conformation. Therefore, the His-tagged MafA_I_ was employed again. A band of 34 kDa corresponding with monomeric MafA_I_ was detected in the heated samples, whilst a band migrating at an apparent molecular weight of ~270 kDa was revealed with antibodies against the His tag when the samples were not denatured before electrophoresis (Figure 5(d)). A similar heat modifiability was detected when MafA_II_ was analyzed (Figure 5(d)); in this case, the anti-MafA_II_ antiserum detected the complex. These results indicate that the MafA proteins form oligomeric complexes. The complexes were also detected when only MafA_I_ or only MafA_II_ was overexpressed from plasmid in the triple Δ*mafA* mutant (results not shown), suggesting the proteins form independent complexes. Purification of the MafA_II_ complex from Coomassie-stained gels and subsequent MALDI-TOF mass spectrometry analysis revealed that the complex was constituted only of MafA protein (Table S2). Together, these results demonstrate that MafA is located in the OM and that it forms a homo-oligomeric complex.

### Dissemination of Maf systems among Gram-negative bacteria

So far, MafA and MafB proteins have only been reported to be present in *Neisseria* spp. and to be particularly abundant among the pathogenic *Neisseria* [10,11]. We performed new BLAST searches and found that the Maf systems are actually much more widely disseminated among Gram-negative bacteria. For example, when using MafA_II_ as a query in protein BLASTs, we identified homologs also in other genera of the *Neisseriaceae*, such as the proteins encoded by the genes with locus tags CX403_RS04975 from *Bergeriella denitrificans* isolate R981, which shows 99.4% sequence identity, or NCTC13336_01484 from *Kingella potus* strain NCTC13336, which shows 94.1% sequence identity. However, homologs were also detected in species from the γ subdivision of Proteobacteria, such as the proteins encoded by the genes with locus tags A9Z60_03095 in *Moraxella nonliquefaciens* strain CCUG60284 (59% identity; E-value 4e^−126^) or BECKTC1821D_GA0114238_101426 in *Candidatus Kentron sp. TC* isolate BECK_BZ123 (49% identity; E-value 1e^−83^). In all these isolates, the *mafA* gene was flanked by a *mafB* gene, characterized by the DUF1020. Homologs were also detected in the δ-proteobacteria, such as the protein encoded by the gene with locus tag Q362_RS19135 from *Desulfobulbus elongatus* strain DSM2908 (E-value 6e^−76^) and even in the Planctomycetes, such as the protein with Seq. ID HBG28899.1 from *Phycisphaerales bacterium* DDX75_17575 (E-value 2e^−17^). However, in these latter cases, the *mafA* homolog is not flanked by a *mafB* homolog; hence, it is not clear whether in these cases the MafA homolog is involved in secretion or, perhaps, only functions as an adhesin. A full account of the dissemination of Maf homologs will be presented elsewhere.

## Discussion

Recently, we and others showed that MafB is a toxin involved in interbacterial competition [10,11]. Jamet *et al*. (2015) concluded that MafB is secreted [11], as judged from the detection of a full-length form of the MafB_I_ protein in the spent culture medium, but they failed to find evidence for an involvement of MafA in the secretion mechanism, i.e. MafB_I_ was also detected in the culture medium when it was heterologously expressed in a *Neisseria cinerea* strain lacking any *mafA*. They proposed that MafB could be released into the medium in OMVs. However, they did not test this possibility, nor did they investigate whether MafB was included inside or exposed on the surface of such OMVs. In our experiments, various MafB_I_ forms, corresponding to the mature protein and the separate DUF1020, were present in the culture medium of a *mafA* mutant, where they were found to be associated with OMVs. Protease-accessibility experiments showed that these polypeptides are localized inside the OMVs, and we hypothesize that this was also the case for the MafB_I_ detected in the culture medium of *mafA*-deficient *N. cinerea* by Jamet *et al*. [11]. Only when MafA was expressed, we detected smaller MafB fragments in the culture medium, which, considering their reactivity with the antiserum used, corresponded with the N-terminal part of the DUF1020 domain. These smaller fragments were not associated with OMVs, and they were not detected by Jamet *et al*. [11], probably because the authors used antibodies against a C-terminal FLAG tag and against a C-terminal MafB peptide.

Reconstitution of the secretion process in *E. coli* unequivocally confirmed the MafA-dependency of MafB translocation across the OM. When produced in the absence of MafA, MafB_III_ was found in the cell lysates and not in the spent medium, consistent with results presented by Jamet *et al*. [11]. Protease-accessibility assays showed that the protein was not exposed on the cell surface in this case. When MafA_II_ was co-expressed, MafB_III_ was still found in the cell lysates, but it was protease accessible, hence transported to the cell surface. This result also demonstrates that MafA is the only neisserial protein required for the secretion of MafB, although we cannot exclude the possibility that a commonly present OM protein, such as BamA, is also involved in the secretion process.

**Figure 6.**
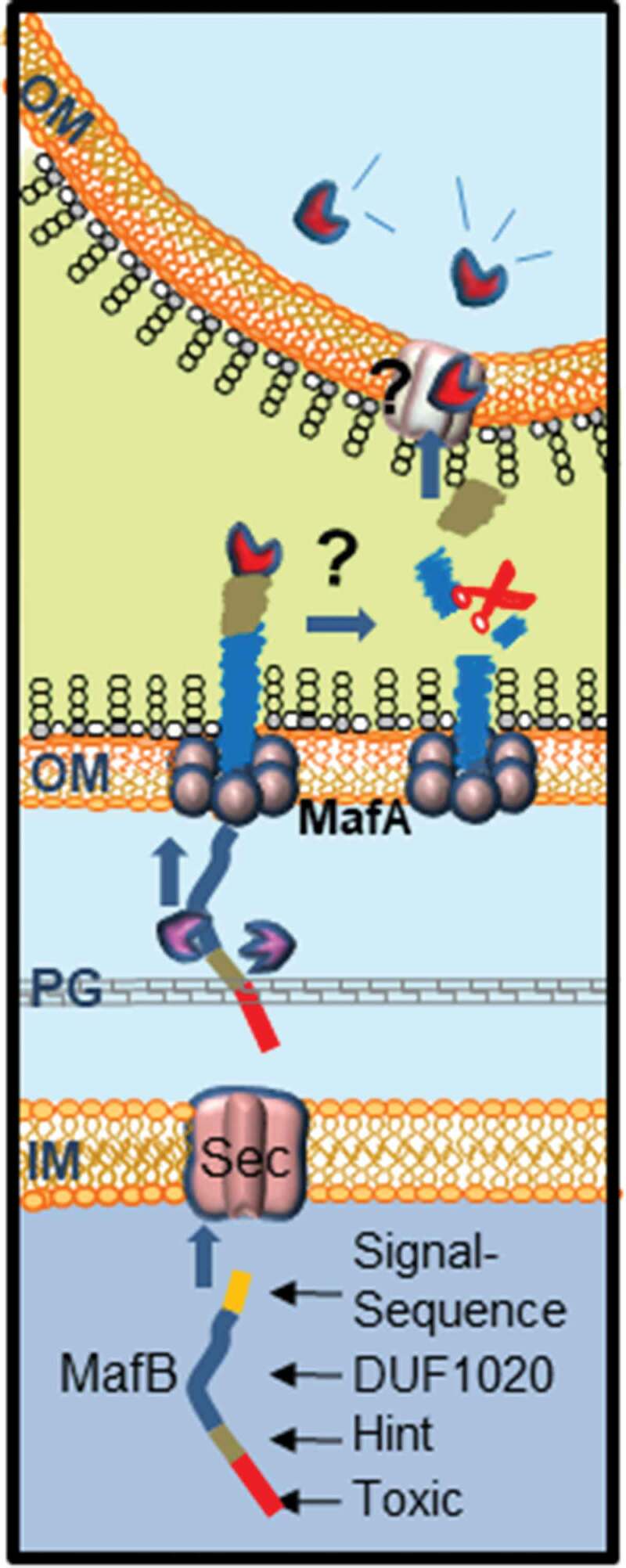
Proposed model for MafB secretion and activation. The MafB proteins contain a cleavable N-terminal signal sequence (yellow) for translocation across the inner membrane (IM) via the Sec translocon. After periplasmic transit, possibly escorted by chaperones like SurA or Skp (purple), MafB interacts with the OM-integrated MafA protein, presumably through its DUF1020 domain. The oligomeric MafA protein constitutes the channel in the OM through which MafB is translocated. After secretion, MafB presumably remains associated with the OM until it interacts with a receptor at the target cell. Then, MafB is cleaved by an unknown protease or via an autocatalytic process that is induced by conformational changes in the protein upon receptor interaction. The C-terminal domain of MafB is thereby released and imported into the target cell where it exerts its toxic activity

Our model for secretion and processing of MafB is presented in Figure 6. MafB proteins are produced with an N-terminal signal sequence followed by a DUF1020 domain, which are both required for the secretion process. The N-terminal signal sequence marks them for transport across the inner membrane to the periplasm probably via the Sec system [11]. In the periplasm, presumably protein chaperones prevent folding of MafB [3]. Then, MafB interacts via the DUF1020 domain with the OM-inserted MafA complex, which mediates its transport through the OM. Our results evidence the coexistence of at least two independent MafA/B secretion systems. MafA_II_ exclusively mediates the secretion of MafB_III_, which is encoded on the same MGI-3 (Figure 1). In contrast, MafA_I_ allows for the secretion of both MafB_I_ and MafB_III_. Possibly, MafA_I_ is also involved in the secretion of MafB_II_. The gene for MafB_II_ is not immediately adjacent to a *mafA* gene, but there is a MafA_I_ encoded on the same MGI (Figure 1), and the DUF1020 domain of MafB_II_ is more related to that of MafB_I_ than to that of MafB_III_ [10]. Similar features were described in meningococcal TPS systems, where the TpsB1 transporter is very specific and recognizes exclusively the cognate TpsA substrates, whilst TpsB2 can transport all available TpsA substrates [28,29]. Like TpsA proteins that function in growth inhibition [5], surface-exposed MafB probably interacts with a receptor on a target cell, after which the C-terminal toxic domain is cleaved and imported into the target cell. Cleavage probably takes place within the DUF1020 domain, and the N-terminal fragments are released into the medium. The proteolytic enzyme that cleaves MafB is not known. Probably, cleavage is an autocatalytic process that is induced by conformational changes in the protein upon receptor interaction, but some MafB proteins also contain a classical Hint domain (i.e. MafB_I_), which may facilitate the release of the C-terminal toxic domain to the medium. Cell-surface-exposed MafB_III_ was not cleaved in *E. coli*, presumably because *E. coli* cells do not express the appropriate receptor.

To function as a transporter for MafB, MafA should be incorporated into the OM. Indeed, we showed that MafA is localized in the OM as an oligomer. Considering the size of the monomer (34 kDa) and the apparent molecular weight of the oligomer (270 kDa), MafA could form a complex constituted of approximately eight subunits. MafA proteins are supposedly lipoproteins and have both α-helical and β-sheet content according to secondary structure prediction (Figure S1a), which is atypical for integral OM proteins. These properties are reminiscent of those of CsgG, an OM protein that transports amyloids, known as Curli, to the cell surface [30,31]. Also, CsgG is a lipoprotein with a mixed α-helical and β-sheet content. Its native structure is a 36-stranded β-barrel composed of nine subunits, where each subunit contributes four β-strands to the barrel, while the α-helices extend into the periplasm [30,31]. Possibly, MafA forms a similar structure. Alternatively, it may be embedded in the OM like the capsular polysaccharide transporter Wza, which also is a lipoprotein [26], and the VirB10 component of type IV secretion system [27]. The transmembrane segments of these oligomeric OM proteins are amphipathic α-helices instead of amphipathic β-strands as found in classical OM proteins. However, helical wheel projections did not indicate that the α-helices in MafA are amphipathic (Figure S1a).

Over the last decade, various contact-dependent growth-inhibition systems have been identified and characterized [32]. These systems are broadly distributed among bacterial species. In general, the toxins produced in these systems are delivered to the target cell utilizing the TPS [5] or Type VI protein secretion system [33]. Both systems can also deliver the toxin into mammalian cells contributing to virulence of pathogens [34,35]. The MafB proteins were recently discovered as a new family of toxic proteins in *Neisseria* spp [10,11]. Here, we demonstrated that these proteins are transported across the bacterial OM employing the MafA proteins. Superficially, the MafA/B systems may look similar to the TPS systems in that a single OM protein is required for the secretion of a toxic module, which is secreted as a part of a larger protein. However, the differences between the systems are large. First, unlike the TpsB proteins, MafA is not an Omp85-family member as it lacks the characteristic POTRA and β-barrel domains. Also, whilst the TpsB proteins function as monomers, MafA proteins form large oligomers. Second, whilst the toxic modules of TpsA and MafB proteins are similar, the rest of these proteins is very different. The TpsA proteins are large filamentous proteins with a β-helical structure presenting typical hemagglutinin repeats and, at the N terminus, a TPS domain required for recognition by the POTRA domains of TpsB [2,29]. All those characteristics are missing in the MafB proteins, which are much smaller and contain a characteristic DUF1020 domain, which is presumably required for recognition by MafA. Thus, the MafA/B system represents a novel secretion system. Interestingly, whereas this system had, so far, only been reported in *Neisseria* spp., we detected homologs now also in other bacterial species. As it has been demonstrated that MafA contributes to the pathogenicity of *Neisseria spp* [14–16]., it will be interesting now to investigate whether the MafA/B system also contributes to the virulence of the pathogenic *Neisseria* by delivering MafB toxins into host cells as described for other CDI systems [35].

## Supplementary Material

Supplemental Material

Supplemental Material
