# Supplementary material for: The outer-membrane protein MafA of Neisseria meningitidis constitutes a novel protein secretion pathway specific for the fratricide protein MafB
Source: Virulence. 2020 Dec 14;11(1):1701–15. doi: 10.1080/21505594.2020.1851940 (PMC7738311; doi:10.1080/21505594.2020.1851940)
Supplement: Supplemental Material [file KVIR_A_1851940_SM5238.docx]

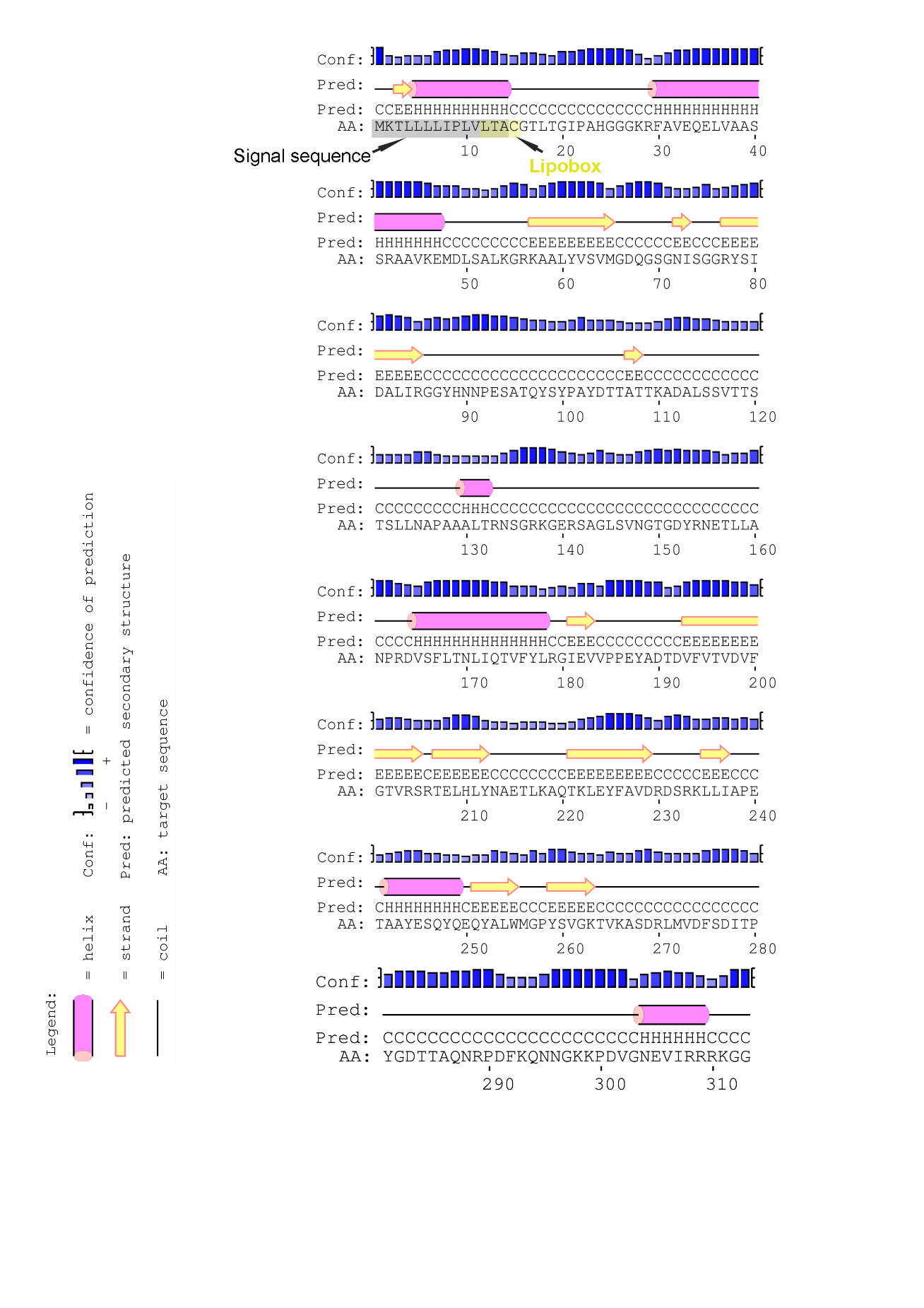
**a**

Residues 30 -47

Residues 165 -178


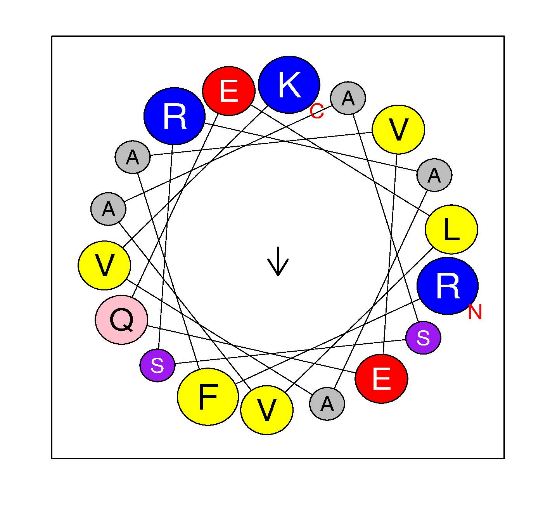

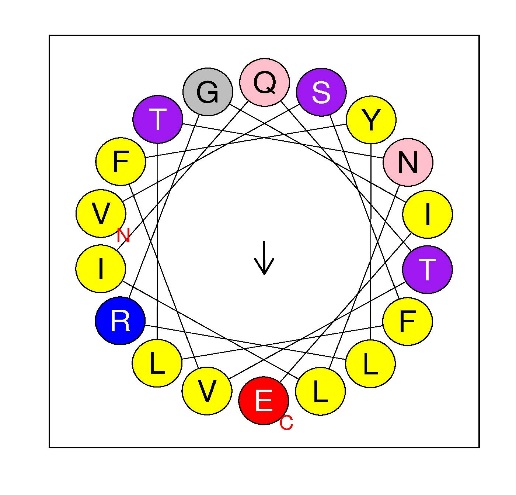


**b**

**
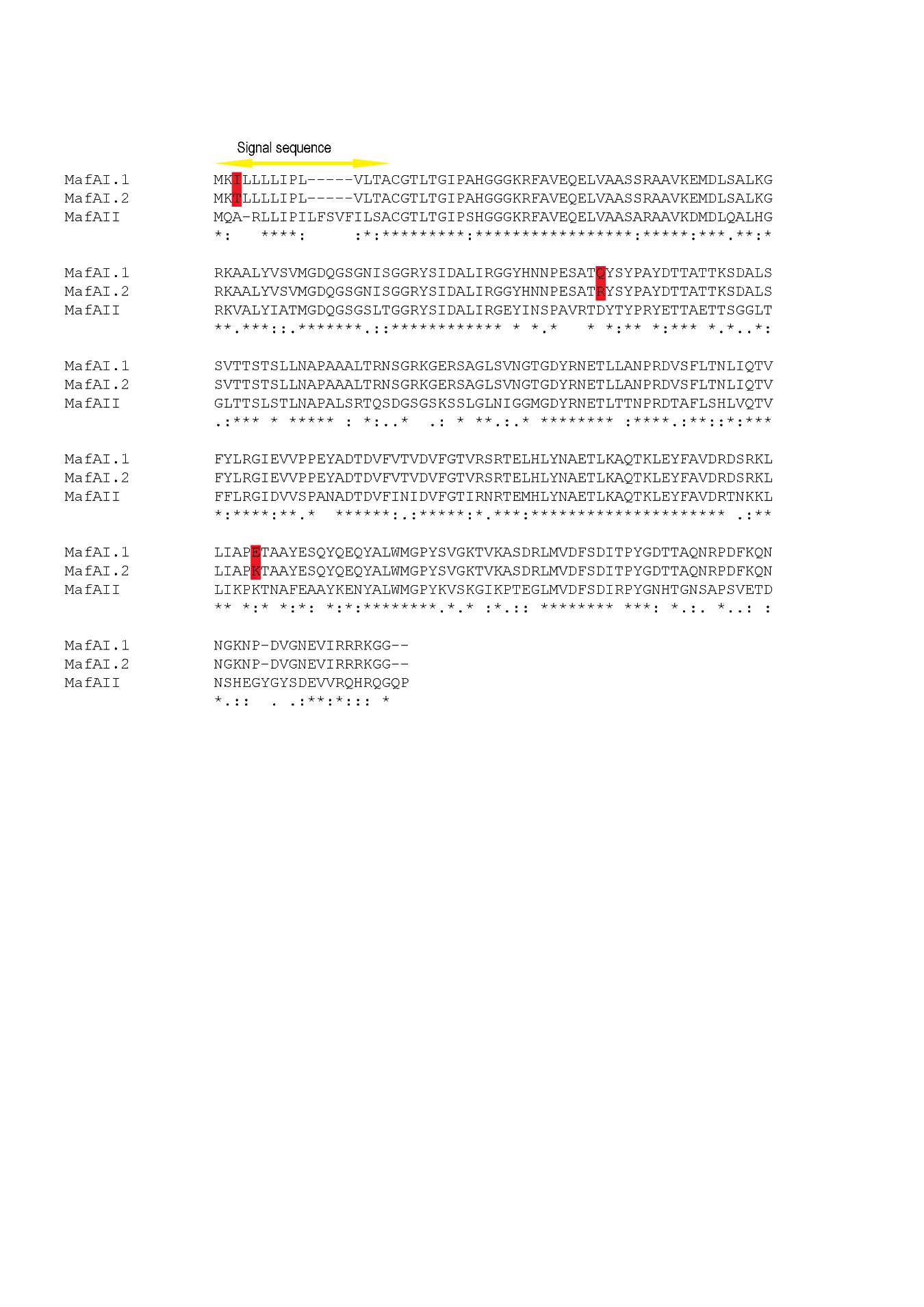
**

**Figure S1.** Sequence and predicted secondary structure of MafA protein. **(a)** Secondary structure prediction for MafA_I_ protein of B16B6 with PsiPred. The typical signal sequence for Sec-mediated translocation with a lipobox motif at the C terminus and ending with a cysteine as the first residue of the mature protein are indicated. Helical wheel predictions of the two major predicted α-helices are depicted at the bottom of the panel. Residues in yellow indicate non-polar amino acids **(b)** Alignment of predicted MafA proteins of BB-1. Residues that are different between the two MafA_I_ proteins are indicated with red color shading, and identical residues in all three aligned proteins are indicated with asterisks.


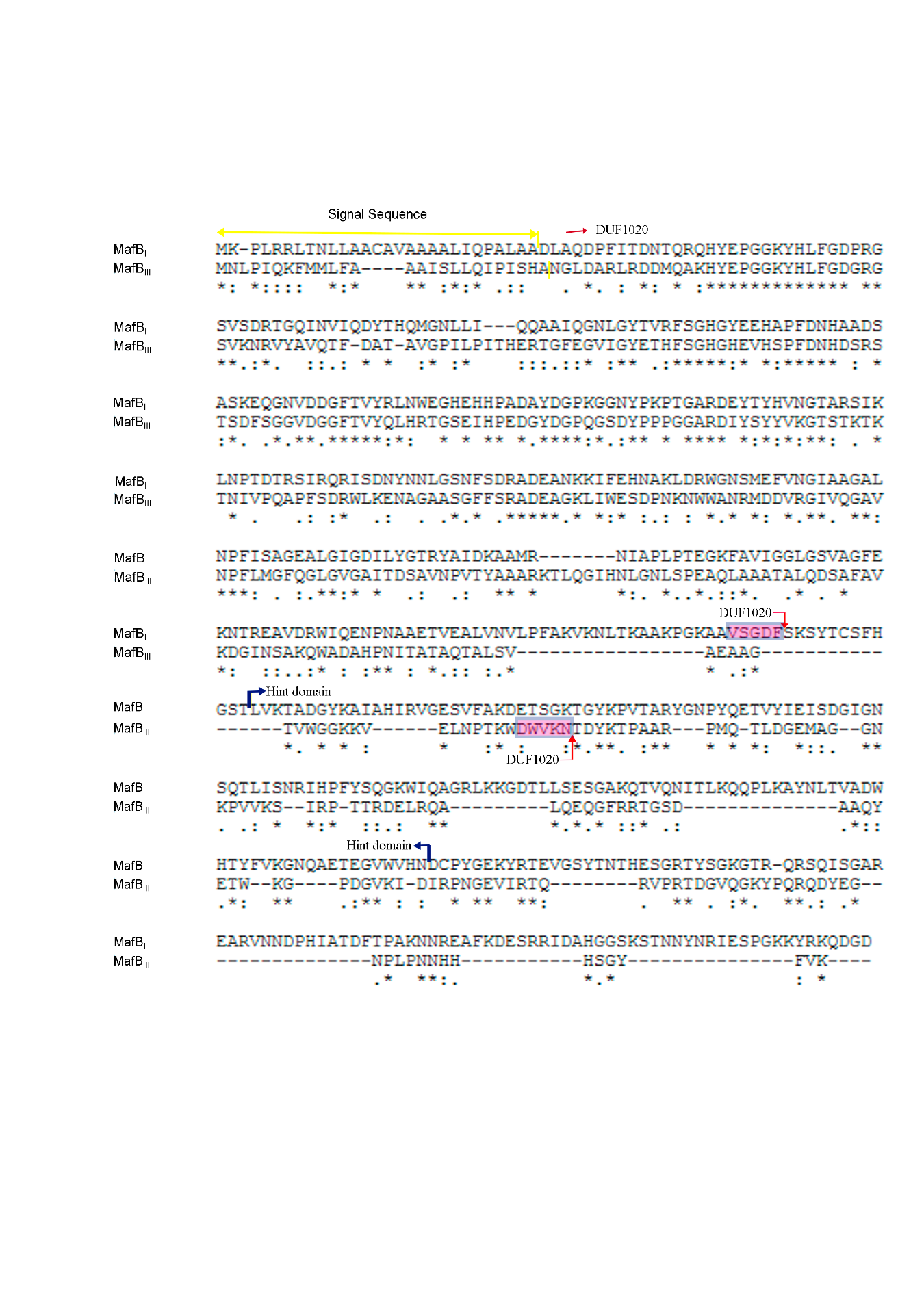


**Figure S2.** Alignment of predicted protein sequences of MafB_I_ and MafB_III_ proteins of FAM18. The alignment was generated with Mafft software. Identical residues in the two aligned proteins are indicated with asterisks. The typical signal sequence for Sec-mediated translocation is indicated. A previously identified conserved VSGDF motif and DWVKN motif [11] in MafB_I_ and MafB_III_ proteins, respectively, limiting the DUF1020 domain, is indicated in a red box. A Hint domain is present in the MafB_I_ protein. The toxic module is located at the variable C-terminal region.


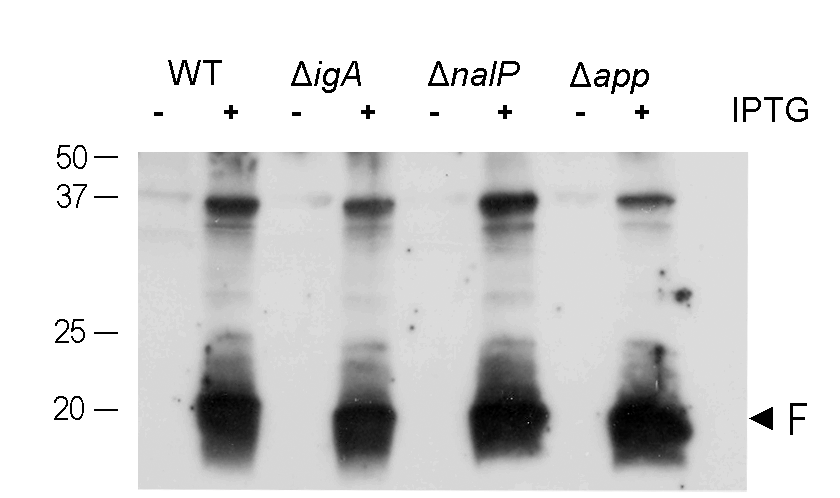


Figure S3. Lack of activity of NalP, IgA protease and App on MafB_I_ cleavage. pEN-*mafB*_I-S_ was introduced in BB-1 (WT) and its derivatives with mutations in *nalP* (Δ*nalP*), *app* (Δ*app*) or *iga* (Δ*iga*). Where indicated, MafB_I_* was overexpressed by addition of IPTG to growth media, and the spent media were analyzed to evaluate the extracellular processing of MafBI. Proteins in spent media were separated by SDS-PAGE and probed on Western blots with antiserum directed against MafB (α-MafB_I_). Arrow-heads indicate proteolytic DUF1020 fragments (F) as described in Figure 2.


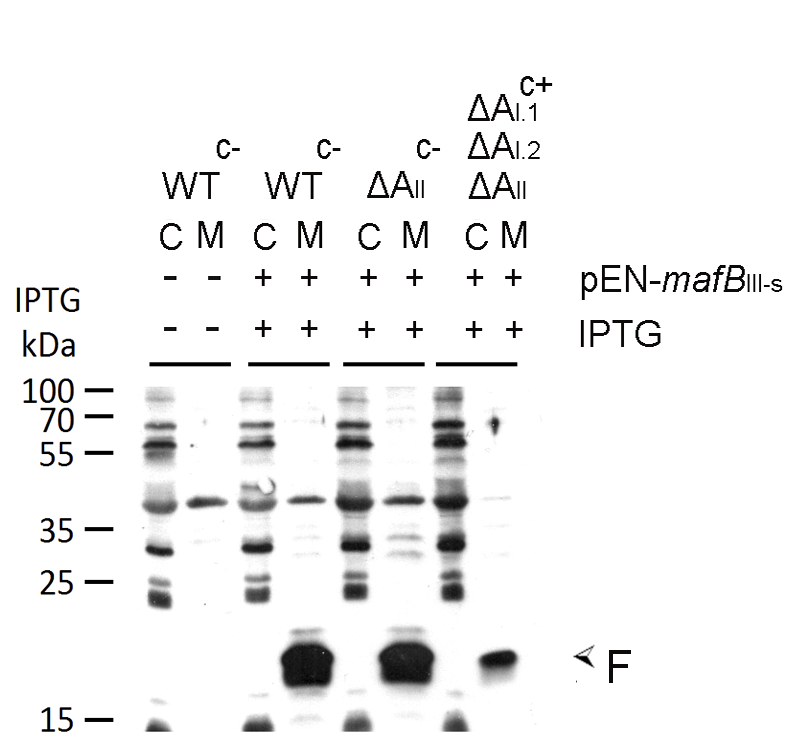


Figure S4. Expression, secretion, and processing of MafB_III_. Proteins in cell lysates (C) and spent medium (M) of the unencapsulated strain BB-1 (WT^c-^), its derivative lacking *mafA*_II_ (ΔA_II_), and a derivative of the encapsulated parental strain B16B6 (^c+^) lacking all *mafA* genes (ΔA_I.1_ΔA_I.2_ΔA_II_) were separated by SDS-PAGE and probed on Western blots with antiserum directed against MafB_III_. The presence of plasmid pEN-*mafB*_III-S_ in the strains and of IPTG during growth is indicated. F indicates proteolytic fragments of MafB_III_. The blot was overexposed (overnight) as compared to Figure 3, to better visualize the presence/absence of MafB_III_-specific bands in cells and spent medium of the strains tested.


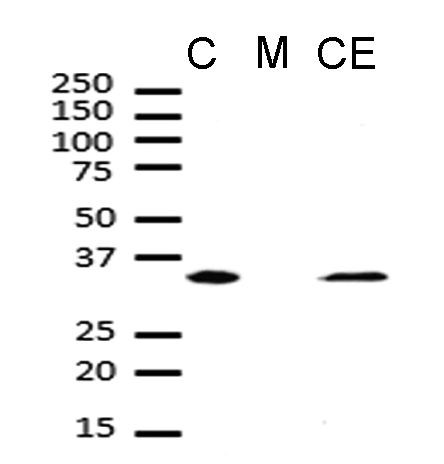


**Figure S5.** Subcellular localization of MafA_I_. The localization of MafA_I_ protein was examined by analyzing cell lysates (C), spent media (M) and cell envelopes (CE) of BB-1 (WT) on Western blots.
