# Supplementary material for: The outer-membrane protein MafA of Neisseria meningitidis constitutes a novel protein secretion pathway specific for the fratricide protein MafB
Source: Virulence. 2020 Dec 14;11(1):1701–15. doi: 10.1080/21505594.2020.1851940 (PMC7738311; doi:10.1080/21505594.2020.1851940)
Supplement: Supplemental Material [file KVIR_A_1851940_SM5237.pdf]

Table S1. Primers used in this study.

Goal PCR	Primer name	Sequence (5'->3')[a]	Remarks
	Preparation of plasmids for overexpression in E. coli		
pET-MafA$_{I\text{-}H}$	MafA$_{I\text{-}}$ ss Fw	CGCGCGCATATGACACTGACCGGCATACCCGCCC	NdeI
	MafA$_{I\text{-}H}$ Rv	GCGCGCGGATCCTTATCCTCCTTTGCGGCGGCGGATGA	BamHI
pET-MafA$_{II\text{-}H}$	MafA$_{II\text{-}}$ss Fw	GCGCGCCATATGACACTGACAGGTATTCCATCG	NdeI
	MafA$_{II\text{-}H}$ Rv	GCGCGCGGATCCTCAAGGTTGCCCTTGTCTATGTTGTCGCA	BamHI
pET-MafB$_{I\text{-}H}$	MafB$_{I\text{-}}$ss Fw	GCGCGCCATATGGCGGACTTGGCGCAAGACCCG	NdeI
	MafB$_{I\text{-}H}$ Rv	GCGCGCGGATCCTTAAAAGGGGTTGAGTGCTC	BamHI
pET-MafB$_{II\text{-}H}$	MafB$_{II\text{-}}$ss Fw	GCGCGCCATATGAACGGTTTGGATGCCCGTTTGC	NdeI
	MafB$_{II\text{-}H}$ Rv	CGCGCGGGATCCTCACATTAAAAAAGGATTAACCGC	BamHI
pIN-B$_{III}$-I	Fw MafBI2	GCGCGCGCTAGCATGAATTTGCCTATTCAAAAATTCA	NheI
	Rv Maf BI2	GCGCGCCGTACGCTATTTTCCCAGTGGCTCAAA	BsiWI
pIN-A$_{II}$-B$_{III}$-I	Fw MafA	GCGCGCGCTAGCATGCAAGCACGGCTGCTGATAC	NheI
	Rv MafABI2	GCGCGCCGTACGCTATTTTCCCAGTGGCTCAAA	BsiWI
	Preparation of plasmids for overexpression in Neisseria		
pEN-*mafB*$_{I\text{-}H}$	Fw MafB$_{I.1}$	CGCGCGCATATGAAACCGCTGCGAAGACTGACA	NdeI
	Rv MafB$_{I.1\text{-}H}$	GCGCGCGGACGTCTCA*ATGATGATGATGATGATGATG* *ATGATGAT*GACAATCATTATGAACCCAAA	AatII
pEN-*mafB*$_{I\text{-}S}$	Fw MafB$_{I.1}$	CGCGCGCATATGAAACCGCTGCGAAGACTGACA	NdeI
	Rv MafB$_{I.1\text{-}S}$	CGCGCGGACGTCTCA*CTTCTCGAACTGCGGGTGGC* *TCCA*ACAATCATTATGAACCCAAA	AatII
pEN-*mafB*$_{III\text{-}S}$	Fw MafB$_{III}$	CGCGCGCATATGAATTTGCCTATTCAAAA	NdeI
	Rv MafB$_{III\text{-}S}$	CGCGCGGACGTCTCA*CTTCTCGAACTGCGGGTGG* *CTCCA*ACCAGCCATTTCCCCATCTA	AatII
pEN-*mafA*$_{I\text{-}H}$	FwMafA$_{I.1}$	GCGCGCCATATGAAAATCCTGCTCCTCCTCATCCC	NdeI
	RvMafA$_{I.1\text{-}H}$	GCGCGCGACGTCTTA*ATGATGATGATGATGATG*T CCTCCTTTGCGGCGGCG	AatII
pEN-*mafA*$_{II\text{-}H}$	FwMafA$_{II}$	GCGCGCCATATGCAAGCACGGCTGCTGAT	NdeI
	RvMafA$_{II\text{-}H}$	GCGCGCGACGTCTTA*ATGATGATGATGATGATG*A GGTTGCCCTTGTCTATGTTG	AatII
	Exchange of resistance cassettes in knock-out constructs		
gm	Fw *gm*	GCGCGCGTCGACGACGCACACCGTGGAAA	SalI
	Rv *gm*	GCGCGCGTCGACGCGGCGTTGTGACAATTT	SalI

[a] Restriction sites used for cloning (underlined) and His-tag and Strep-tag sequences (in italics) included in primers are indicated.

Table S2. MASCOT search results obtained by MALDI-TOF analysis of the MafA$_{II}$ complex of *N. meningitidis**.

**Observed**	**Mr (expected)**	**Mr (calculated)**	**ppm[1]**	**Start-end**	**Miss[2]**	**Ions**	**Peptide**
915.4296	914.4223	914.4134	9.77	100 - 106	0		R-TDYTYPR-Y
915.4296	914.4223	914.4134	9.80	100 - 106	0		R-TDYTYPR-Y
950.5408	949.5335	949.5233	10.8	82 - 89	0		R-YSIDALIR-G
1012.5244	1011.5171	1011.5025	14.4	227 - 234	0		K-LEYFAVDR-T
1012.5244	1011.5171	1011.5025	14.4	227 - 234	0	18	K-LEYFAVDR-T
1105.5723	1104.5650	1104.5564	7.85	90 - 99	0		R-GEYINSPAVR-T
1390.7418	1389.7345	1389.7252	6.71	34 - 46	0		R-FAVEQELVAASAR-A
1390.7418	1389.7345	1389.7252	6.71	34 - 46	0	44	R-FAVEQELVAASAR-A
1440.7533	1439.7460	1439.7409	3.59	223 - 234	1		K-AQTKLEYFAVDR-T
1540.8011	1539.7938	1539.7827	7.20	47 - 60	1		R-AAVKDMDLQALHGR-K + Oxidation (M)

*Mass 34817. Score = 138. Protein scores greater than 83 are significant (P<0.05). Identification: MafA adhesin. MafA protein was identified in different Neisseria strains. Only data for *Neisseria meningitidis* FAM18, which belongs to the same clonal complex that B16B6, are shown. [1] Error of the calculated Mass expressed as parts per million. [2] Number of miss cleavage sites within the peptide.
